# Publication trends of artificial intelligence in retina in 10 years: Where do we stand?

**DOI:** 10.3389/fmed.2022.1001673

**Published:** 2022-11-02

**Authors:** Jingyuan Yang, Shan Wu, Rongping Dai, Weihong Yu, Youxin Chen

**Affiliations:** ^1^Department of Ophthalmology, Peking Union Medical College Hospital, Chinese Academy of Medical Sciences and Peking Union Medical College, Beijing, China; ^2^Key Laboratory of Ocular Fundus Diseases, Chinese Academy of Medical Sciences and Peking Union Medical College, Beijing, China; ^3^Beijing Hospital, National Center of Gerontology, Institute of Geriatric Medicine, Chinese Academy of Medical Sciences, Beijing, China

**Keywords:** artificial intelligence, bibliometric, deep learning, retina, retinal diseases

## Abstract

**Purpose:**

Artificial intelligence (AI) has been applied in the field of retina. The purpose of this study was to analyze the study trends within AI in retina by reporting on publication trends, to identify journals, countries, authors, international collaborations, and keywords involved in AI in retina.

**Materials and methods:**

A cross-sectional study. Bibliometric methods were used to evaluate global production and development trends in AI in retina since 2012 using Web of Science Core Collection.

**Results:**

A total of 599 publications were retrieved ultimately. We found that AI in retina is a very attractive topic in scientific and medical community. No journal was found to specialize in AI in retina. The USA, China, and India were the three most productive countries. Authors from Austria, Singapore, and England also had worldwide academic influence. China has shown the greatest rapid increase in publication numbers. International collaboration could increase influence in this field. Keywords revealed that diabetic retinopathy, optical coherence tomography on multiple diseases, algorithm were three popular topics in the field. Most of top journals and top publication on AI in retina were mainly focused on engineering and computing, rather than medicine.

**Conclusion:**

These results helped clarify the current status and future trends in researches of AI in retina. This study may be useful for clinicians and scientists to have a general overview of this field, and better understand the main actors in this field (including authors, journals, and countries). Researches are supposed to focus on more retinal diseases, multiple modal imaging, and performance of AI models in real-world clinical application. Collaboration among countries and institutions is common in current research of AI in retina.

## Introduction

The application of artificial intelligence (AI) in retinal images has shown reliable performance as well as or better than human clinicians at some key medical care tasks, such as analysis of images, diagnosis, and prediction of prognosis (1). Currently ophthalmologists begin to embrace an age of AI-assistant ophthalmology. However, how to evaluating and applying AI technique in retina and to integrate AI technique into ophthalmic profession remain challenging. Analyzing previous works is helpful to identify current research situation in AI in retina.

Due to these issues, a comprehensive review of the publications in AI in retina is urgently needed. Bibliometric analyses are helpful to address these problems by describing distribution patterns of publications, geographical distribution of research, latest evolution in the field of AI in retina. Therefore, bibliometrics is helpful in understanding of a specific field and in governing policymaking (2). Bibliometric analysis is a sort of original studies and not a systematic review or meta-analysis. However, to our knowledge, no similar studies which focused on AI in retina have been specifically conducted. Consequently, there is a lack of knowledge about the research situation in the field of AI in retina. In this study, we investigated the frontier researches of and the trends within the fields of AI in retina across the international scientific literature. We also tried to predict trends for the next few years, noting that the increase of the amount of AI researches in retina is expected to lead to a better application of AI technique in real-world settings.

## Materials and methods

### Search methods

Web of Science (WOS) Core Collection is regarded as the most suitable database for bibliometric analysis. The search for papers to be included in the current study was carried on July 4, 2022, and all the included publications were published from January 1, 2012 to July 1, 2022. The search strategy was “(TS = artificial intelligence OR TS = deep learning) AND (TS = retina OR TS = vitreous OR TS = choroid)”. 622 literatures were identified. 77 publications were excluded except articles and review articles according to the document type, and 6 non-English publications were also excluded. 599 publications were included ultimately.

### Data collection

All the data were extracted and downloaded from WOS databases, including metrics of publication numbers, countries and regions, authors, citations, and H-indexes. The classification of countries and regions were defined according to the default classification in Web of Science. The literatures from Hong Kong were part of the literatures from People’s Republic of China (China). We also investigated the relationship between global productivity of AI in retina and Human development index (HDI), which measures the level of human development based on knowledge, life expectancy, and income per capita indicators, rather than economic growth alone. Human development report 2020 was published by United Nations Development Programme (3). Countries and areas were divided into four categories based on HDI, including very high human development, high human development, medium human development, and low human development. The countries and regions classification system for Human development index, which were come up with by United Nations, were converted to the classification in Web of Science. Prism 9, R (R. app GUI 1.79), VOSviewer 1.6.18, and SPSS 26 were used to input and analyze data.

### Bibliometric analysis

The descriptive indexes were extracted from WOS and calculated by SPSS. The co-occurrence networks of keywords, authors, and countries/regions were constructed by VOSviewer. The keywords were extracted from titles and abstracts. Frequencies over 20 was the criteria of the inclusion for analyses. Average appearing year was used to assess the novelty of keywords. For creating the wordcloud of keywords, Biblioshiny, an R tool, was used to generate wordcloud map of keywords. Frequencies over 20 was also the criteria of the inclusion for analyses. H-indexes were collected from WOS database, and can partially reflect the impact of researchers. Relative research interest (RRI) was defined as the number of publications in a specific field per year divided by all publications in all fields per year (formula in Supplementary Table). The value of this metric reflects the global attention and study interest in a specific field. A higher value of RRI for AI in retina represents more research interest and more research hotspots in this field. The third order polynomial method was used in the prediction model using Prism. In order to analyze the increasing trend of publication numbers, we calculated the average growth rate, compound average growth rate, relative growth rate, and doubling time (formulas in Supplementary Table). For investigating the degree of international collaboration, the degree of collaboration was calculated (formula in Supplementary Table), and Pearson’s correlation of publication numbers among countries was calculated.

## Results

### Productivity and collaboration between countries and regions

**FIGURE 1:**
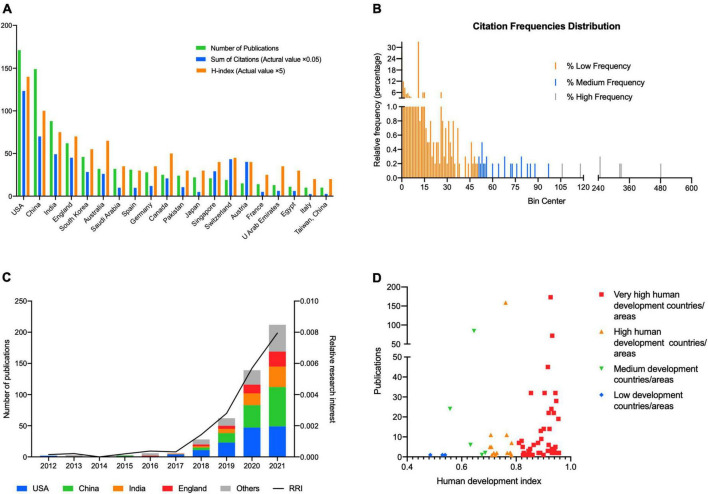
**(A)** Top 20 countries/regions in the publications of artificial intelligence in retina. The green bar shows the number of publications, the blue bar shows the sum of citations in total (actual value multiply by 0.05), and the orange bar shows the H-index (actual value multiply by 5). **(B)** The relative frequency (percentage) distribution of publications in various citation number. The publications were divided into three groups according to the total citation frequency, including high citation frequency (more than 100 citations) group, medium frequency (more than 50 citations and < 100 citations) group, and low frequency (<50 citations) group. **(C)** The proportion of publications of USA, China, India, England, and other countries/regions and relative research interest (RRI) in each year on the field of artificial intelligence in retina. **(D)** The number of publications of artificial intelligence in retina in countries or areas of various levels of human development. Very high human development countries or areas contributed to most publications.

A total of 599 publications were analyzed. From 2012 to 2022, USA contributed to the most publications (171, 28.5%), followed by China (149, 24.9%) (Figure 1A). More than half publications were contributed by USA and China. Except USA and China, no countries published more than 100 publications. The total number of publications on AI in retina has maintained steady growth over the past 10 years, especially in the past 5 years (Figure 1C). USA and China were also the countries with highest H-index, and the top 5 counties with most publications also had the highest H-index. USA also published the most papers per year from 2012 until now, and published 45 papers. Additionally, RRI of AI in retina also increased from < 0.001% in 2012 to 0.008% in 2021 (Figure 1C), indicating that the research interest of AI in retina keep increasing worldwide over the past decade. According to the HDI category, we noticed that most publications were from very high human development countries or areas, and the numbers of publications of AI in retina were consistent with HDI classification on the whole (Figure 1D).

We analyzed the co-occurrence of 32 countries and regions (Supplementary Figure 1); the analysis suggested 6 clusters: 1. USA, and Taiwan, China; 2. China, and South Africa; 3. India, Suadi Arabia, Pakistan, U Arab Emirates, Egypt, Bangladesh, Poland, and Russia; 4. South Korea, Japan, France, Malaysia, and Vietnam; 5. England, Spain, Germany, Iran, Brazil, Italy, Turkey, and Israel; and 6. Australia, Singapore, Canada, Switzerland, and Austria, Netherlands.

The top 10 countries/regions of high degree of collaboration were Singapore (95.2%), England (87.1%), Germany (85.7%), Saudi Arabia (84.4%), Switzerland (84.2%), Pakistan (79.2%), Australia (75.0%), Canada (68.0%), South Korea (67.4%), and USA (63.7%). Although China contributed a large number of publications, the degree of collaboration of China was only 39.6%. We further investigated the correlation among countries and regions, and found no significant correlation was detected between countries (all *P* value > 0.1).

**FIGURE 2:**
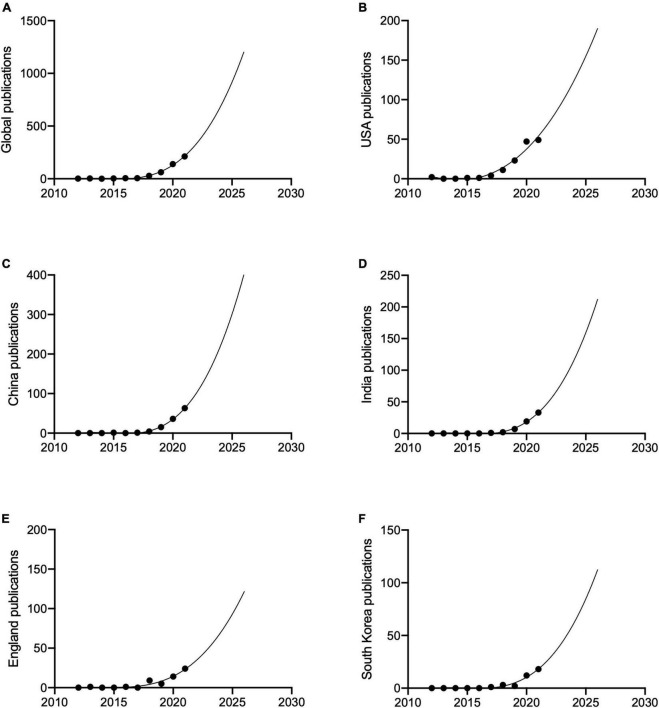
The publication trends and prediction curve of global and countries which had the most publications since 2012. **(A)** Global. **(B)** USA. **(C)** China. **(D)** India. **(E)** England. **(F)** South Korea.

The publication rate of papers on AI in retina has continued to increase over the past 10 years; predictions for next 5 years show this increase continuing (Figure 2). China has shown the greatest rapid increase in publication numbers since 2012. USA is projected to maintain its leading position and steady growth, but has the possibility to publish less papers than China and India in 2025.

For global publication number from 2012 to 2021, the average growth rate was 79.4%, compound average growth rate was 67.9%, relative growth rate was 81.2%, and doubling time was 1.3 years.

### Citations and H-index

WOS citation reports revealed a total of 6445 citations without self-citations of the 7267 relevant citations since 2012. Each paper was cited an average of 12.13 times. USA contributed to the most citations (2466 citations, 2366 without self-citations) and H-index (28) (Figure 1A) from 2012. China ranked second in both citations and H-index (1401 citations, 1339 without self-citations, H-index 20).

The most cited publication has been cited for a total of 481 times (Figure 1B). We divided these publications into three groups according to the frequencies, including high frequency (more than 100 citations), medium frequency (more than 50 citations and < 100 citations), and low frequency (< 50 citations). Most of the publications were in low frequency group, 24 papers were cited with a medium frequency and only 7 papers were cited with a high frequency.

To further explore the distribution of citation number in each year, we supplemented the heatmaps of each group of citation frequency (Supplementary Figures 2B–D). Every row in the heatmap represents a publication, the x axis means year, and the color represents the citation number. The time span of high frequency and medium frequency is similar, and is longer than that of most publications in low frequency group. Besides, we also analyzed the distribution of publication year of each group (Supplementary Figure 2A). Most of the publications in low frequency group were published in recent years, and that could possibly due to less citations.

### The leading institutions, journals and authors

**FIGURE 3:**
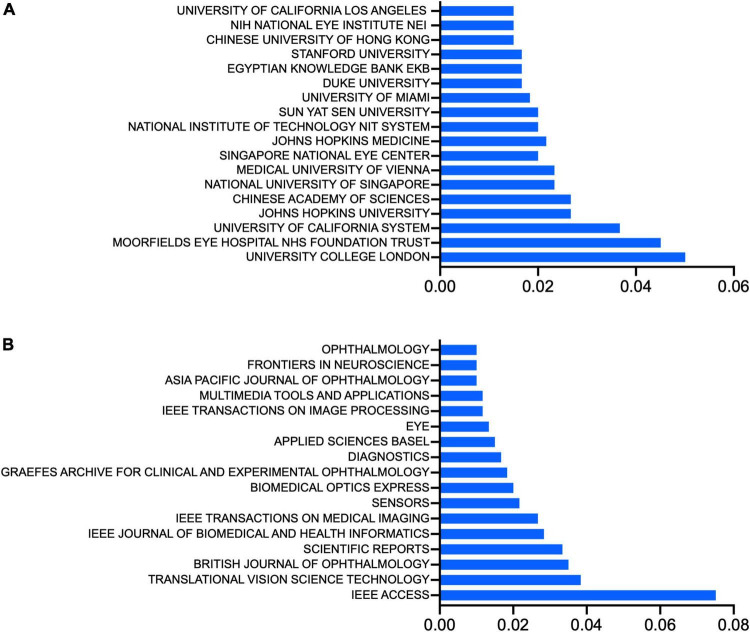
**(A)** Top institutions, ranked by the percentage of their publications in the total number of publications of artificial intelligence in retina. **(B)** Top journals, ranked by the percentage of their publications in the total number of publications of artificial intelligence in retina.

We examined the top institutions in this field and found that the University of College London (30, 5.01%), and the Moorfields Eye Hospital NHS Foundation Trust (27, 4.51%) in England published the most papers on AI in retina since 2012. University of California system (22, 3.67%), Johns Hopkins University (16, 2.67%), and Chinese Academy of Sciences (16, 2.67%) ranked third and fourth (Figure 3A).

About half (287, 47.91%) of the papers on AI in retina were published in 27 journals, including *IEEE Access*, which published the most relevant publications (45). *Translational Vision Science Technology* and *British Journal of Ophthalmology* and published the second- and third-most with both 21 publications (Figure 3B).

The 10 papers with the most citations in total are displayed in Table 1. The most cited paper was published in *IEEE Transactions on Medical Imaging*, a classic and authoritative medical imaging periodical, and is called *Segmenting Retinal Blood Vessels With Deep Neural Networks*. The corresponding author was Pawel Liskowski. Most publications of AI in retina were published in journals focusing on engineering and computing (Table 2).

**TABLE 1:** The top 10 papers with the most citations relevant to artificial intelligence in retina.

Title	Corresponding authors	Journal	Publication year	Total citations
Segmenting retinal blood vessels with deep neural networks	Liskowski, P	IEEE T MED IMAGING	2016	481
A reconfigurable on-line learning spiking neuromorphic processor comprising 256 neurons and 128K synapses	Indiveri, G	FRONT NEUROSCI-SWITZ	2015	328
Artificial intelligence and deep learning in ophthalmology	Ting, DSW	BRIT J OPHTHALMOL	2019	323
Artificial intelligence in retina	Schmidt-Erfurth, U	PROG RETIN EYE RES	2018	247
Real-time classification and sensor fusion with a spiking deep belief network	Pfeiffer, M	FRONT NEUROSCI-SWITZ	2013	247
Retinal vessel segmentation based on fully convolutional neural networks	Oliveira, A; Silva, CA	EXPERT SYST APPL	2018	118
Using a deep learning algorithm and integrated gradients explanation to assist grading for diabetic retinopathy	Peng, L	OPHTHALMOLOGY	2019	106
Multi-categorical deep learning neural network to classify retinal images: A pilot study employing small database	Yoo, TK; Rim, TH	PLOS ONE	2017	97
A deep learning ensemble approach for diabetic retinopathy detection	Shamshirband, S	IEEE ACCESS	2019	88
Choroid segmentation from optical coherence tomography with graph edge weights learned from deep convolutional neural networks	Zheng, YJ	NEUROCOMPUTING	2017	85

**TABLE 2:** Top 10 Web of Science categories of journals on artificial intelligence in retina research.

Web of Science categories	No. of publications (%)
Ophthalmology	133 (22.20)
Engineering Electrical Electronic	123 (20.53)
Computer Science Information Systems	93 (15.53)
Computer Science Artificial Intelligence	70 (11.69)
Radiology Nuclear Medicine Medical Imaging	60 (10.02)
Computer Science Interdisciplinary Applications	59 (9.85)
Telecommunications	56 (9.35)
Engineering Biomedical	53 (8.85)
Mathematical Computational Biology	41 (6.85)
Multidisciplinary Sciences	35 (5.84)

The top 10 authors in this field are listed in Table 3 according to the number of publications and citations, as well as their position in author orders. The works of Ursula Schmidt-Erfurth from Medical University of Vienna were published the most since 2012, with 9 papers and 458 citations (449 without self-citations). Hrvoje Bogunoviæ, also from Medical University of Vienna, ranked second with 8 publications and 464 citations (460 without self-citations). Pearse A. Keane also ranked third with 8 publications and 368 citations (364 without self-citations) (Table 3).

**TABLE 3:** Top 10 authors who published most and cited most in the field of artificial intelligence in retina.

Author	Country	Latest Affiliation	No. of publications	No. of citations
Schmidt-Erfurth U	Austria	Medical University of Vienna	9	455
Bogunovic H	Austria	Medical University of Vienna	8	464
Keane PA	England	Moorfields Eye Hospital NHS Foundation Trust	8	368
Balaskas K	England	Moorfields Eye Hospital NHS Foundation Trust	7	75
Gerendas BS	Austria	Medical University of Vienna	7	383
Lee AY	USA	University of Washington	7	410
Wong, Tien Y	Singapore	National University of Singapore	6	401
Yoo TK	South Korea	Aerosp Med Ctr	6	124
Cheung Carol Y	China	Chinese University of Hong Kong	6	99
Huang TJ	China	Peking University	6	63

We also analyzed cooperation between investigators (Supplementary Figure 3); the node size within a collaboration network indicates the strength of the connections between every author. Several authors, including David Alonso-Caneiro, Michael J. Collins, Scott A Read, and Ursula Schmidt-Erfurth had close cooperation to other researchers and teams. The researchers in the figure usually lead large-scale research teams and maintain close connections to others in the field of AI in retina.

### Research hotspots in artificial intelligence in retina

**FIGURE 4:**
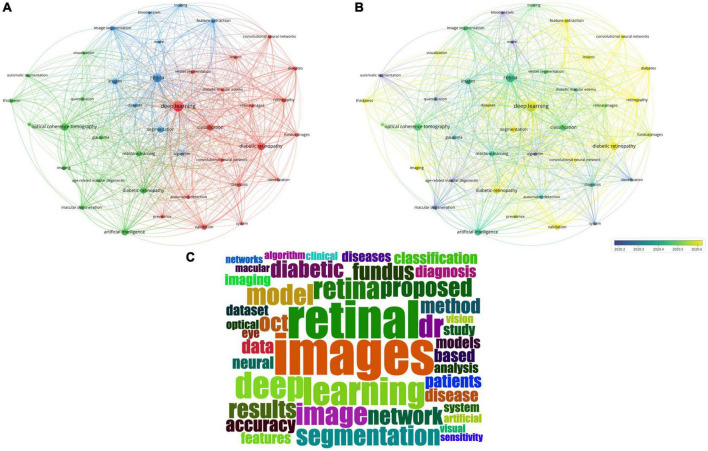
Keywords analysis by VOSviewer and R. **(A)** Co-occurrence map of keywords in titles and abstracts. Keywords were classified into three clusters by co-occurrence frequency, including diabetic retinopathy-related cluster (in red), optical coherence tomography on multiple diseases-related cluster (in green), algorithm-related cluster (in blue). **(B)** Color-coded map of keywords by the average time of appearance. Blue keywords appeared earlier, while yellow keywords appeared more recently. **(C)** The wordcloud map the most frequent keywords.

Keywords analysis defined the most frequently used words and their linkage within the field of AI in retina research. We analyzed the keywords that appeared over 20 times across the included publications. Merging repeated words excluded meaningless ones resulted in 41 total keywords that can be divided into three primary clusters by co-occurrence frequency, including diabetic retinopathy-related cluster (in red), optical coherence tomography (OCT) on multiple diseases-related cluster (in green), algorithm-related cluster (in blue) (Figure 4A).

We also color-coded the keywords by average time of appearance, and found that these keywords appeared in a short period (Figure 4B). And the words “retinal images” and “deep learning” were the most frequent words (Figure 4C).

## Discussion

The current study aims to conduct a bibliometric analysis related to AI in retina. We found that the increasing trends in AI in retina since 2012. USA has published the most publications (45 publications), the most citations (1976 citations in total) and the highest H-index (24). China shows the greatest potential on publications in this field. The authors who published most on AI in retina come from Austria, England, USA, Singapore, South Korea, and China. By analyzing keywords, we summarized previous hotspots and predicted future hotspots. We found that AI algorithm studies, diabetic retinopathy-related AI studies, OCT-related AI studies, and other application studies of AI on retina are the current hotspots in the field of AI in retina. The average years of appearance for keywords are concentrated over a relatively short period, which suggests that AI in retina is an attractive topic in science. These results suggest the rapid progress made in AI in retina research, which might guide the research directions of future studies.

In the current study, we searched publications in the last decade, which were published since 2012. The current mainstream of artificial intelligence network was deep learning network (4), which was found to be most suitable for imaging data and replaced classic machine learning (1). Gulshan et al. and Ting et al. reported the application of deep learning network on detection of diabetic retinopathy based on fundus photographs achieved high sensitivity and specificity, respectively (5, 6). From then on, more and more papers focused on deep learning network on retina were published, which was accord with our results. The deep learning networks presented satisfied outcomes and potential to revolutionize how ophthalmology is practiced in the future (7). Various deep learning technologies have been applied in this field, such as generative adversarial networks and automated machine learning (8, 9). Current AI models mainly rely on cloud computing, which usually requires high bandwidth and low latency. With the development of communication technology, such as 5G, local edge models or offline AI models that could run on compact low power devices and do not require a continuous internet connection could help alleviate some of application challenges, and improve the quality of healthcare in underdeveloped areas (10–12). The trend of rapid development of AI in retina occurs in recent years. Therefore, we included publications in the latest decade (from only 2 publications in 2012 to 212 publications in 2021), rather than much earlier studies, whose methods might not suitable for current medical imaging tasks in most settings. And we used *deep learning* as topic words rather than other AI methods to search studies. Moreover, as this scientific research field is extremely dynamic (13), in order to include most recent publications, we included 138 publications in the first half of 2022, just before we wrote the manuscript. Therefore, considering the development of the field of AI in retina and its rapid progress, we included relevant publications only published in the latest decade.

In the field of AI in retina, the metric of RRI has maintained growth over the last 10 years from < 0.001% in 2001 to 0.004% in 2021, and the number of publications also increased rapidly. These indicate great interest in AI in retina on the part of the scientific and medical community. It is noticed that the number of publications in AI in retina grew more and more sharply after 2016 (total number of publications was more than 98% in the present study). In the year when Google published its influential work of AI in detection of diabetic retinopathy (5), Microsoft announced that 2016 would be the year of AI (14). This shows that the current wave of interest in AI covers many fields beyond ophthalmology. However, the current percentage of articles related to AI in retina published in the same journals which published most relevant papers was not high, and these papers were scattered in a great number of journals (more than 100 journals). The lack of a specialized journal could lead to difficulties for researchers to track latest developments in AI in retina.

When analyzing countries and authors, we found that the countries which published most papers were not consistent with the countries which had most influential authors. For example, USA (171 publications) and China (149 publications) contributed to the greatest number of publications, but only 1 author in the top 5 authors with greatest number of citations came from USA. On the contrary, authors from Austria and Singapore had the top worldwide influence according to the number of citations. On one hand, except benefiting from the bonus of number of researchers, the analysis of co-occurrence of countries and regions revealed that researchers in USA and China tend to collaborate with researchers from other countries (Supplementary Figure 1). On the other hand, this position of Singapore and Austria could be explained not only by the production of local institutions, such as National University of Singapore (14 publications) and Medical University of Vienna in Austria (14 publications), but also by the willing of collaboration (13, 15). Another possible reason is that researchers in small countries have much more cooperation partners outside of their countries than partners in their countries (16). International collaboration on AI-related studies has shown its advantages on offering opportunities to countries regardless of country area (17). Furthermore, we applied a classification system based on HDI, and found that most of the top countries (8 of top 10 countries)and top authors (8 of top 10 authors) belongs to the level of very high human development countries. Therefore, cooperation and collaboration between countries and areas of various levels of human development are needed to promote the popularity of AI technique. However, the unavoidable filling of collaboration agreements and negotiation on intellectual property issues with partners on an institutional level might cause loss of interest and risk of postponing cooperation due to prolonged preparation before onset of AI researches (18). Researchers might need to investigate a new rapid pattern for international collaboration.

Besides the effort from medical researchers and AI engineers, a concerted effort from all stakeholders, including administrations, patients, and insurances, is needed. Sustainable models of AI applications in real world worth further investigation, taking the benefit of the patient, the health care provider, and the payer into consideration (18). Ethical and legal regulations for AI researches, which usually involve data of patient privacy (19), are also needed to cover the full workflow of AI studies and clinical trials.

We analyzed the top journals which published most papers and top articles which were most cited, and found that most publications of AI in retina are technical researches related to technology, engineering, and computing (6 of top 10 journals), not related to clinical practice. Similar to most AI researches in the category of ophthalmology, researches of AI in retina mainly focus on technical methods (7 of top 10 publications) (13). The analysis revealed that this research field involves more scientists rather than clinicians. In the future, more efforts are supposed to be paid on translation of the AI technique into a powerful tool in clinical practice on screening, triage, and decision-making process (20).

Based on the analysis of keywords in the current studies, most studies of AI in retina are correlated with diabetic retinopathy (200 relevant publications in the current study) (5, 6), followed by optical coherence tomography and associated technology (172 relevant publications). AI has also been used in diagnosing age-related macular degeneration (60 relevant publications) (21, 22), glaucoma (57 relevant publications) (23), and retinopathy of prematurity (15 relevant publications) (24). However, the technique of AI could be used for assistance for screening and triage of more retinal diseases, such as retinal detachment (14 relevant publications) and retinal vein occlusion (8 relevant publications), which are also vision-threatening diseases. More studies are needed for diagnosis of multiple retinal diseases simultaneously, and even general conditions (25), which meets the current clinical needs (26).

Moreover, multimodal imaging method rather than single modal imaging method is currently mainstream in real-world settings. However, most AI studies used single modal images of fundus photography or optical coherence tomography as training data, and the performance of trained models usually cannot overcome the challenges from numerous variabilities in reality, including field of view, image magnification, image quality, and race origin (7). Only limited researches investigated the application of multimodal imaging in AI researches, which not only improved the diagnostic performance, but also a more precise definition (27). Considering the growing demand of healthcare and limited number of retinal specialists, particularly in rural areas, the application of AI on more retinal diseases and multimodal imaging could help enhance medical care for multiethnic populations in real-world clinical practice.

We analyzed the keywords and abstracts of current studies of AI in retina, and found that most studies focused on imaging analysis (436 relevant publications), such as segmentation and detection of lesions, and diagnosis. Recently, Ting et al. found that using AI system as an assistive tool to screen for diabetic retinopathy is an economical method in real-world settings (28). The technique of AI could also be used for guidance of therapy with automated detection of lesion activity, quantitative analysis of therapeutic effects, and determination of recurrence (1). However, most studies of AI in retina were conducted in a well-prepared setting with selected data, and the performance of AI models in real world might showed unexpected outcomes. A team from Google used a deep learning system in clinics for detection of diabetic retinopathy (29), and failure to reproduce the performance they published in 2016 due to socio-environmental factors (5). Moreover, increase the diversity of dataset by applying training data from various sources helps enhance the generalization ability of AI models, and helps bring AI models to clinical practice (30). Therefore, the validation of application of AI technique in real world is an important topic in future researches.

Although studies of AI in retina is broad, it mainly focuses on detecting structures and lesions and diagnosing diseases from fundus photography and optical coherence tomography images through deep learning algorithms currently, trying to achieve expert-level performance in well-prepared experiment environment. Enhancing the performance of detecting retinal diseases in real world in encouraged (29). Firstly, additional structured clinic data (including but not limited to, age, sex, ethnicity, other examination results, medical history, clinical diagnosis, comorbidities, or genetic indicators, etc.) or multimodal images could be collected together. This would be helpful to build datasets with diversity and further to cover more application scene and more populations, such as screening and clinical diagnosis in countries and regions of various development levels. Secondly, more application requirements from clinical practice were put up with, including earlier detection and referral, personalized treatment based on guidelines and the reality of each patient and prediction of prognosis, covering more diseases for global automated screening, resulting in lower economic burden and better life quality for patients. AI has the potential to provide direct patient care after combining with mobile devices and communication technology, especially in under-resourced areas (31). However, there is a lot of work to be done to make this a reality, and the application of AI models in clinical practice needs clinical validation and regulatory requirements. Moreover, AI models are supposed to progress in parallel with the advance of new management strategies.

Our study has several limitations. The nature of selection bias existed in the methods, including in favor of English-language journal and only papers published on authoritative and influential journals which were listed in WOS Core Collection were included in our analysis. Although most high-quality papers are included by most databases, the results might be partially affected. Moreover, using different searching method in WOS Core Collection could lead to different results, and the method we used did not include publications that were assigned same keywords by other databases, which would not result in unrepeatable searching results. Therefore, the total number of included publications using forementioned methods might be less than some other methods.

In summary, this study comprehensively analyzed most published researches on AI in retina, and presents a current view of mainstream studies on AI in retina. AI in retina is a very attractive topic in researches, and the relevant technique developed rapidly, although no journal specializes in AI in retina. International collaboration is important in conduction of influential researches. Researches are supposed to focus on more retinal diseases, multiple modal imaging, and performance of AI models in real world. This study may help clinicians, but also scientists understand the current trend of publications of AI in retina, know the main actors in the field, and predict and guide the future developments in this research field.

## Data availability statement

The original contributions presented in this study are included in the article/Supplementary material, further inquiries can be directed to the corresponding author.

## Author contributions

JY: design, definition of intellectual content, data acquisition, data analysis, manuscript preparation, and manuscript editing. SW: design, definition of intellectual content, data acquisition, data analysis, funding, manuscript preparation, and manuscript editing. RD and WY: manuscript review. YC: concepts and manuscript review. All authors contributed to the article and approved the submitted version.

## Supplementary material

Supplementary Figure 1 The co-occurrence map of 32 countries and regions, which showed the international collaboration among countries/regions.

Supplementary Figure 2 **(A)** The distribution of publication year for publications of various citation frequency. **(B–D)** The heatmaps of high citation frequency (more than 100 citations) group, medium frequency (more than 50 citations and < 100 citations) group, and low frequency (<50 citations) group, respectively. Every row in the heatmap represents a publication. The color represents the total citation number in each year (x axis).

Supplementary Figure 3 The co-occurrence map of scholars who published papers of artificial intelligence in retina, which showed the cooperation among researchers.

## Abbreviations

AI: artificial intelligence
HDI: Human development index
OCT: optical coherence tomography
RRI: Relative research interest
WOS: web of science.

## References

[1] Schmidt-Erfurth U Sadeghipour A Gerendas BS Waldstein SM Bogunoviæ H. Artificial intelligence in retina. *Prog Retin Eye Res.* (2018) 67:1–29. 10.1016/j.preteyeres.2018.07.004 30076935

[2] King DA. The scientific impact of nations. *Nature.* (2004) 430:311–6. 10.1038/430311a 15254529

[3] UNDP. *Human Development Report 2020.* New York, NY: UNDP (2020).

[4] LeCun Y Bengio Y Hinton G. Deep learning. *Nature.* (2015) 521:436–44. 10.1038/nature14539 26017442

[5] Gulshan V Peng L Coram M Stumpe MC Wu D Narayanaswamy A Development and validation of a deep learning algorithm for detection of diabetic retinopathy in retinal fundus photographs. *JAMA.* (2016) 316:2402–10. 10.1001/jama.2016.17216 27898976

[6] Ting DSW Cheung CY Lim G Tan GSW Quang ND Gan A Development and validation of a deep learning system for diabetic retinopathy and related eye diseases using retinal images from multiethnic populations with diabetes. *JAMA.* (2017) 318:2211–23. 10.1001/jama.2017.18152 29234807 PMC5820739

[7] Ting DSW Pasquale LR Peng L Campbell JP Lee AY Raman R Artificial intelligence and deep learning in ophthalmology. *Br J Ophthalmol.* (2019) 103:167–75. 10.1136/bjophthalmol-2018-313173 30361278 PMC6362807

[8] Liu Y Yang J Zhou Y Wang W Zhao J Yu W Prediction of OCT images of short-term response to anti-VEGF treatment for neovascular age-related macular degeneration using generative adversarial network. *Br J Ophthalmol.* (2020) 104:1735–40. 10.1136/bjophthalmol-2019-315338 32217538

[9] Yang J Zhang C Wang E Chen Y Yu W. Utility of a public-available artificial intelligence in diagnosis of polypoidal choroidal vasculopathy. *Graefes Arch Clin Exp Ophthalmol.* (2020) 258:17–21. 10.1007/s00417-019-04493-x 31686211

[10] Merenda M Porcaro C Iero D. Edge machine learning for AI-enabled IoT devices: a review. *Sensors.* (2020) 20:2533. 10.3390/s20092533 32365645 PMC7273223

[11] Keane PA Topol EJ. Medicine and meteorology: cloud, connectivity, and care. *Lancet.* (2020) 395:1334. 10.1016/s0140-6736(20)30813-8 32334694

[12] Greco L Percannella G Ritrovato P Tortorella F Vento M. Trends in IoT based solutions for health care: moving AI to the edge. *Pattern Recognit Lett.* (2020) 135:346–53. 10.1016/j.patrec.2020.05.016 32406416 PMC7217772

[13] Boudry C Al Hajj H Arnould L Mouriaux F. Analysis of international publication trends in artificial intelligence in ophthalmology. *Graefes Arch Clin Exp Ophthalmol.* (2022) 260:1779–88. 10.1007/s00417-021-05511-7 34999946

[14] The New York Times blog. *Microsoft Reorganizes Its Research Efforts Around A.I.* New York, NY: The New York Times blog (2016).

[15] Tran BX Vu GT Ha GH Vuong QH Ho MT Vuong TT Global evolution of research in artificial intelligence in health and medicine: a Bibliometric study. *J Clin Med.* (2019) 8:360. 10.3390/jcm8030360 30875745 PMC6463262

[16] Mindeli LE Markusova VA. Bibliometric studies of scientific collaboration: international trends. *Autom Doc Math Linguist.* (2015) 49:59–64. 10.3103/S0005105515020065

[17] Narin F Stevens K Whitlow ES. Scientific co-operation in Europe and the citation of multinationally authored papers. *Scientometrics.* (1991) 21:313–23. 10.1007/BF02093973

[18] Ting DSW Peng L Varadarajan AV Keane PA Burlina PM Chiang MF Deep learning in ophthalmology: the technical and clinical considerations. *Prog Retin Eye Res.* (2019) 72:100759. 10.1016/j.preteyeres.2019.04.003 31048019

[19] Price WN II Cohen IG. Privacy in the age of medical big data. *Nat Med.* (2019) 25:37–43. 10.1038/s41591-018-0272-7 30617331 PMC6376961

[20] Kras A Celi LA Miller JB. Accelerating ophthalmic artificial intelligence research: the role of an open access data repository. *Curr Opin Ophthalmol.* (2020) 31:337–50. 10.1097/icu.0000000000000678 32740059 PMC8095451

[21] Burlina PM Joshi N Pekala M Pacheco KD Freund DE Bressler NM. Automated grading of age-related macular degeneration from color fundus images using deep convolutional neural networks. *JAMA Ophthalmol.* (2017) 135:1170–6. 10.1001/jamaophthalmol.2017.3782 28973096 PMC5710387

[22] Grassmann F Mengelkamp J Brandl C Harsch S Zimmermann ME Linkohr B A deep learning algorithm for prediction of age-related eye disease study severity scale for age-related macular degeneration from color fundus photography. *Ophthalmology.* (2018) 125:1410–20. 10.1016/j.ophtha.2018.02.037 29653860

[23] Li Z He Y Keel S Meng W Chang RT He M. Efficacy of a deep learning system for detecting glaucomatous optic neuropathy based on color fundus photographs. *Ophthalmology.* (2018) 125:1199–206. 10.1016/j.ophtha.2018.01.023 29506863

[24] Brown JM Campbell JP Beers A Chang K Ostmo S Chan RVP Automated diagnosis of plus disease in retinopathy of prematurity using deep convolutional neural networks. *JAMA Ophthalmol.* (2018) 136:803–10. 10.1001/jamaophthalmol.2018.1934 29801159 PMC6136045

[25] Rim TH Lee G Kim Y Tham YC Lee CJ Baik SJ Prediction of systemic biomarkers from retinal photographs: development and validation of deep-learning algorithms. *Lancet Digit Health.* (2020) 2:e526–36. 10.1016/s2589-7500(20)30216-8 33328047

[26] Li B Chen H Zhang B Yuan M Jin X Lei B Development and evaluation of a deep learning model for the detection of multiple fundus diseases based on colour fundus photography. *Br J Ophthalmol.* (2022) 106:1079–86. 10.1136/bjophthalmol-2020-316290 33785508

[27] Morelle O Wintergerst M Finger RP. [Multimodal imaging and evaluation in the age of artificial intelligence] Multimodale bildgebung und -auswertung im zeitalter von künstlicher intelligenz. *Ophthalmologe.* (2020) 117:965–72. 10.1007/s00347-020-01210-6 32845382

[28] Xie Y Nguyen QD Hamzah H Lim G Bellemo V Gunasekeran DV Artificial intelligence for teleophthalmology-based diabetic retinopathy screening in a national programme: an economic analysis modelling study. *Lancet Digit Health.* (2020) 2:e240–9. 10.1016/s2589-7500(20)30060-1 33328056

[29] Beede E Baylor E Hersch F Iurchenko A Wilcox L Ruamviboonsuk P A human-centered evaluation of a deep learning system deployed in clinics for the detection of diabetic retinopathy. In: *Proceedings of the 2020 CHI Conference on Human Factors in Computing Systems. Association for Computing Machinery.* Honolulu HI: (2020). p. 1–12.

[30] Benet D Pellicer-Valero OJ. Artificial intelligence: the unstoppable revolution in ophthalmology. *Surv Ophthalmol.* (2022) 67:252–70. 10.1016/j.survophthal.2021.03.003 33741420 PMC9757817

[31] Bellemo V Lim ZW Lim G Nguyen QD Xie Y Yip MYT Artificial intelligence using deep learning to screen for referable and vision-threatening diabetic retinopathy in Africa: a clinical validation study. *Lancet Digit Health.* (2019) 1:e35–44. 10.1016/s2589-7500(19)30004-4 33323239

