# Supplementary figures and images for: Publication trends of artificial intelligence in retina in 10 years: Where do we stand?
Source: Front Med (Lausanne). 2022 Nov 2;9:1001673. doi: 10.3389/fmed.2022.1001673 (PMC9666394; doi:10.3389/fmed.2022.1001673)

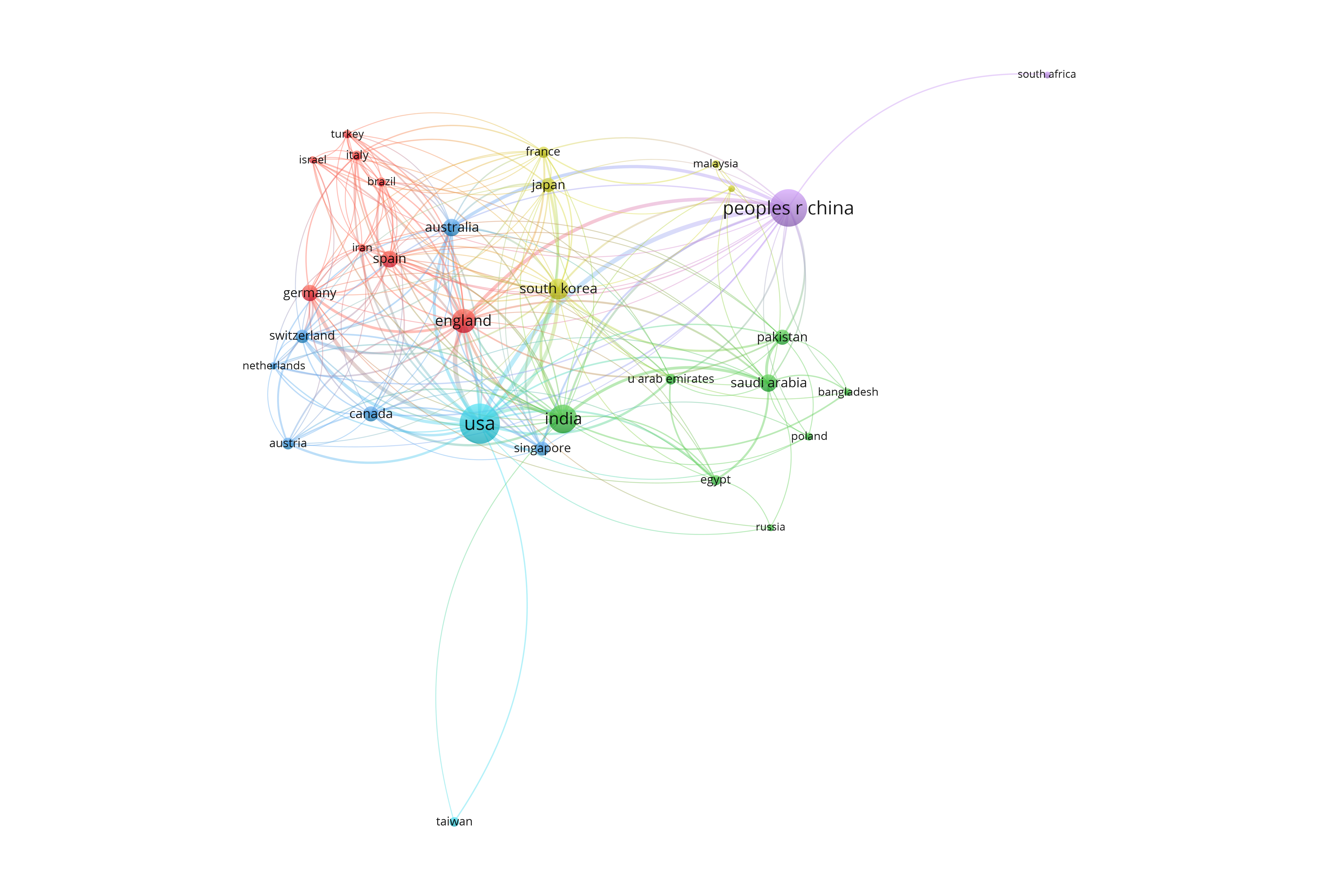

Supplement: Supplementary Figure 1 — The co-occurrence map of 32 countries and regions, which showed the international collaboration among countries/regions. [file Image_1.JPEG]

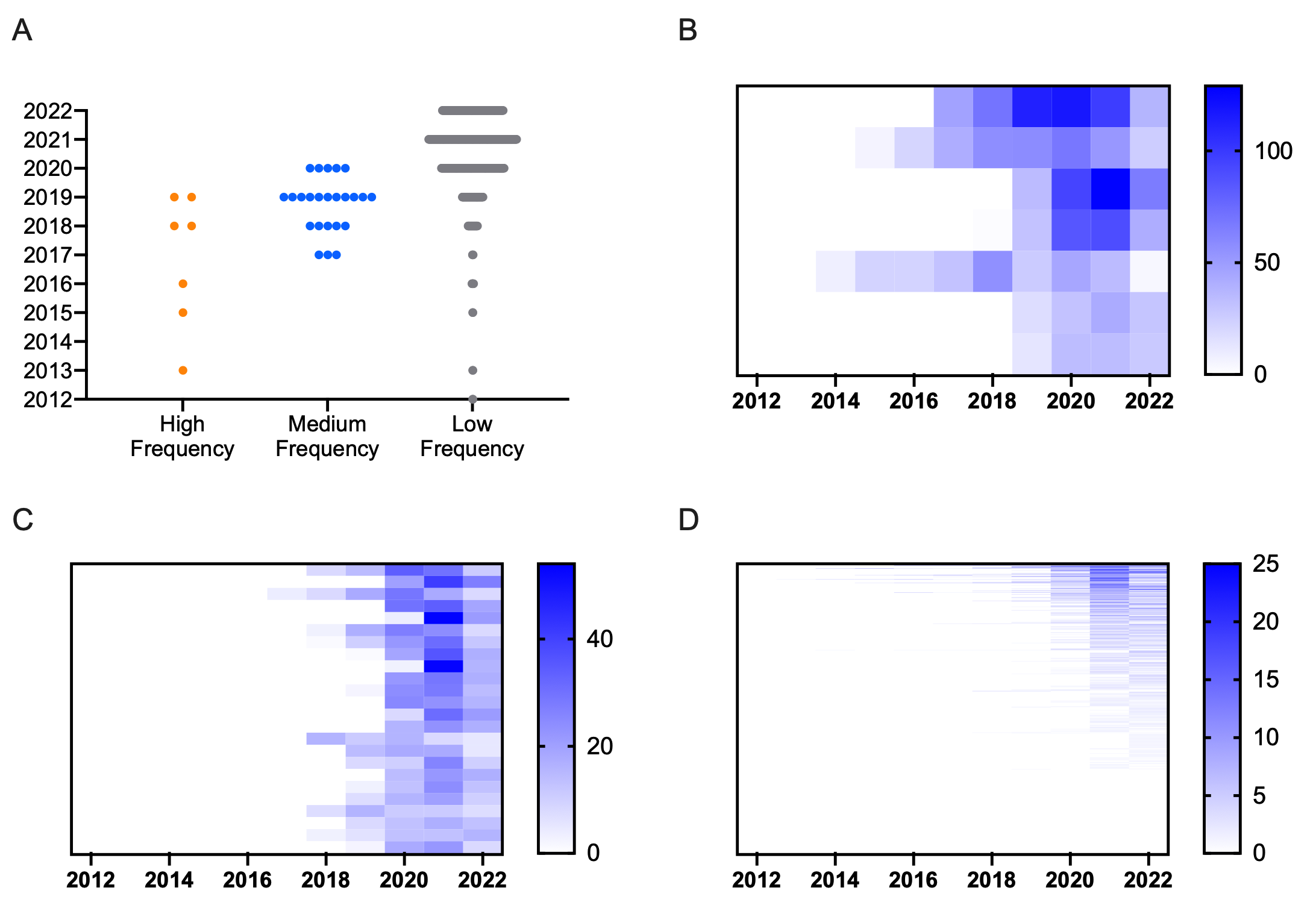

Supplement: Supplementary Figure 2 — (A) The distribution of publication year for publications of various citation frequency. (B–D) The heatmaps of high citation frequency (more than 100 citations) group, medium frequency (more than 50 citations and < 100 citations) group, and low frequency (<50 citations) group, respectively. Every row in the heatmap represents a publication. The color represents the total citation number in each year (x axis). [file Image_2.TIFF]

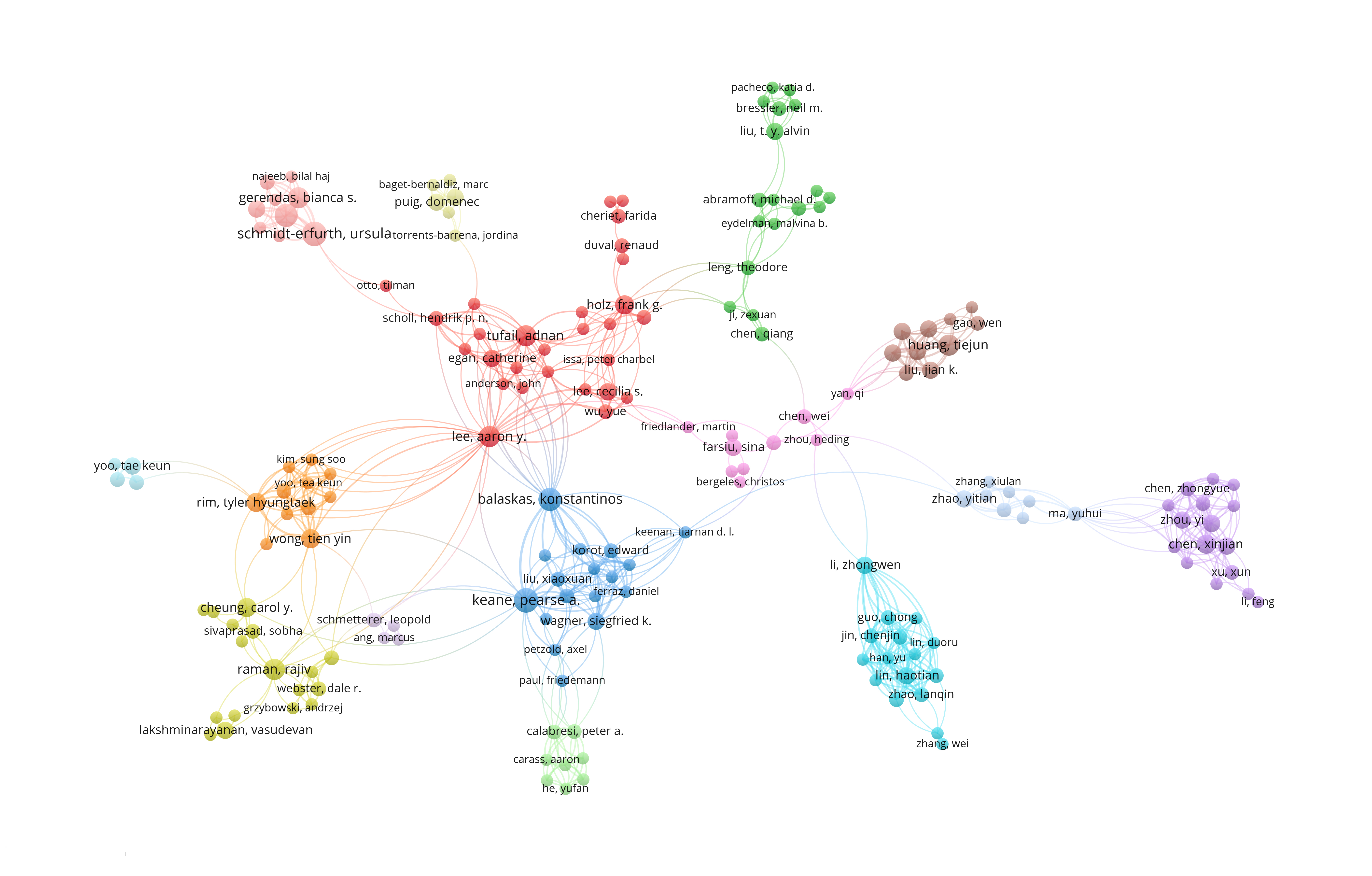

Supplement: Supplementary Figure 3 — The co-occurrence map of scholars who published papers of artificial intelligence in retina, which showed the cooperation among researchers. [file Image_3.JPEG]
