# Supplementary material for: Publication trends of artificial intelligence in retina in 10 years: Where do we stand?
Source: Front Med (Lausanne). 2022 Nov 2;9:1001673. doi: 10.3389/fmed.2022.1001673 (PMC9666394; doi:10.3389/fmed.2022.1001673)
Supplement: Supplementary file 4 [file Table_1.docx]

**Supplementary Table.** Calculating formulas for relative research interest, average growth rate, compound average growth rate, relative growth rate, doubling time, and degree of collaboration.

| Metrics | Formulas used in the current study |
| --- | --- |
| Relative research interest | Publication number in a specific field per year / all publication number in all fields per year |
| Average growth rate | Average value of the growth rate of each year |
| Compound average growth rate | (Publication number in 2021 / publication number in 2012) ^ (1 / (year 2021 - year 2012) - 1 |
| Relative growth rate | ((Publication number in 2021 - publication number in 2012) / publication number in 2012) ^ (1 / (year 2021 - year 2012) |
| Doubling time | (Year 2021 - year 2012) * (log_10_2 / (log_10_(publication number in 2021) – log_10_(publication number in 2012))) |
| Degree of collaboration | The number of publications by at least 2 countries/regions that includes a specific country/region / the number of all publications by the specific country/region |
